# Current Status of Poly(ADP-ribose) Polymerase Inhibitors as Novel Therapeutic Agents for Triple-Negative Breast Cancer

**DOI:** 10.1155/2012/829315

**Published:** 2011-10-25

**Authors:** David J. Hiller, Quyen D. Chu

**Affiliations:** ^1^Department of General Surgery, Wake Forest University School of Medicine, Winston-Salem, NC 27157, USA; ^2^Department of Surgery and Division of Surgical Oncology, Louisiana State University Health Sciences Center in Shreveport and the Feist-Weiller Cancer Center, Shreveport, LA 71130, USA

## Abstract

Triple-negative breast cancer (TNBC) is an aggressive type of breast cancer that is clinically defined as lacking estrogen and progesterone receptors, as well as being ERBB2 (HER-2) negative. Without specific therapeutic targets, TNBC carries a worse prognosis than other types of breast cancer in the absence of therapy. Research has now further differentiated breast cancer into subtypes based on genetic expression patterns. One of these subtypes, basal-like, frequently overlaps with the clinical picture of TNBC. Additionally, both TNBC and basal-like breast cancer link to BRCA mutations. Recent pharmaceutical advances have created a class of drugs, poly(ADP-ribose) polymerase (PARP) inhibitors, which are showing potential to effectively treat these patients. The aim of this paper is to summarize the basis behind PARP inhibitors and update the current status of their development in clinical trials for the treatment of TNBC.

## 1. Introduction

Breast cancer is a multifaceted, heterogeneous disease whose treatment is evolving as genetic profiles shed more light on potential targets. The understanding of breast cancer became more complex with Perou et al.'s 2000 publication detailing the classification of breast cancer based on gene-expression assays [1]. Among this classification was the basal-like subtype, described as frequently (but not always) being ER, PR, and HER-2 deficient while also expressing basal cytokeratins 5/6 and 17 and epidermal growth factor (EGFR) [1, 2]. These basal-like breast cancers make up 17 to 37% of all breast cancers [2–4]. Having genetic profiles outlining the inherent differences in breast cancer has allowed for new research paths attempting to develop novel therapeutics that are subtype dependent. 

The definition of triple-negative breast cancer is based on clinical observations; the tumor must lack estrogen receptors (ERs), progesterone receptors (PRs), and hormone epidermal growth factor receptor type 2 (HER-2) expression. These tumors are particularly vexing for physicians because there are no known endocrine targets nor are there specific receptors to block. Women diagnosed with TNBC tend to be younger [5] and are more likely to present with poorly differentiated tumors [6]. Although TNBC is responsive to chemotherapy and features a higher pathologic complete response (pCR) rate compared to other breast cancer types (in the presence of neoadjuvant therapy) [7], the prognosis for TNBC patients is still poor [7, 8]. 

**Figure 1:**
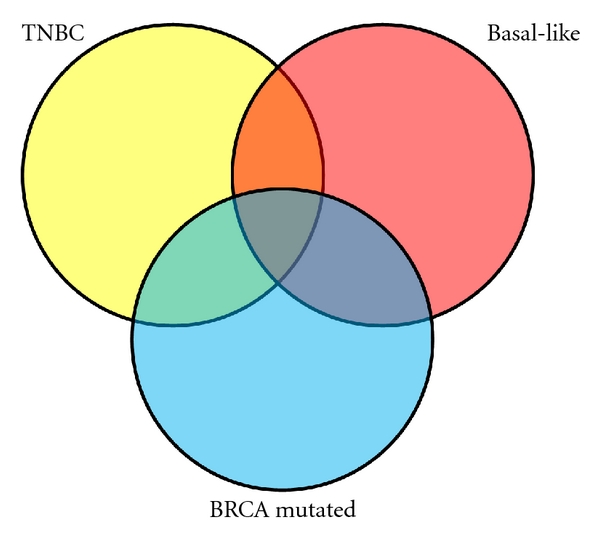
A Venn diagram representing the connection of TNBC, basal-like breast cancer, and BRCA-mutated breast cancer.

There are many similarities between TNBC and basal-like breast cancer, but the two terms are not synonymous (Figure 1). They share demographic characteristics such as age of first menarche and increased incidence in the African-American [9] and Hispanic [10] female population. It has been noted that roughly 80% of TNBC tumors are basal-like breast cancers [11]. However, immunohistochemical studies have shown that 17–40% of basal-like breast cancers do not have a triple-negative phenotype [12]. Up to 20% of basal-like breast cancers actually express ER or HER-2 to some extent [13]. 

One important similarity between TNBC and basal-like breast cancer is the incidence of mutations in the breast cancer susceptibility gene 1 and 2 (BRCA1 and 2). BRCA mutations are only 2-3% of all breast cancers but signify an increased lifetime risk of breast and ovarian cancer [14]. Somatic BRCA mutations or inactivation of the gene can also occur. It is estimated that methylation of the BRCA1 promoter can be found in 11–14% of sporadic breast cancers [15–17]. BRCA1 is a key player in mammary gland development [18], and both BRCA1 and BRCA2 are connected with DNA repair [14]. A majority of tumors in women with BRCA mutations feature similar expression patterns as basal-like tumors [18–20], clouding the picture of where BRCA-mutated cancers, basal-like breast cancers, and TNBC originate (Figure 1).

 Researchers have found the links between TNBC, basal-like breast cancer, and BRCA mutations to be a potential source of directed therapy. One notable avenue is through synthetic lethality. This is a strategy to target and kill specific cell types, without collateral damage. It is achieved by locating a gene that, when inhibited, will kill cancerous cells that contain a specific genetic signature. The inhibitor would not damage normal cells that lack the cancer-specific gene. The design and exploration of poly(ADP-ribose) polymerase (PARP) inhibitors have emerged as a potential target to cause synthetic lethality in cancerous cells while sparing normal mammary tissue. The aim of this paper is to discuss the molecular basis behind PARP inhibitors and an update on their current status in several clinical trials.

## 2. PARP1 Inhibitors

Poly(ADP-ribose) polymerase (PARP) is a nuclear protein that is activated in the presence of DNA damage. While several PARP proteins have been detected, PARP1 and PARP2 have been associated with DNA stability [21]. When single strand DNA (ssDNA) damage occurs, it is identified and repaired by a cellular process that includes PARP and base excision repair [22]. If ssDNA breaks are not repaired (e.g., PARP inhibition), the breaks build up and are converted at the replication fork to double-strand DNA (dsDNA) breaks [23–25]. At this point, homologous recombination or nonhomologous end joining repairs the double-stranded breaks in DNA [23, 25]. 

**Figure 2:**
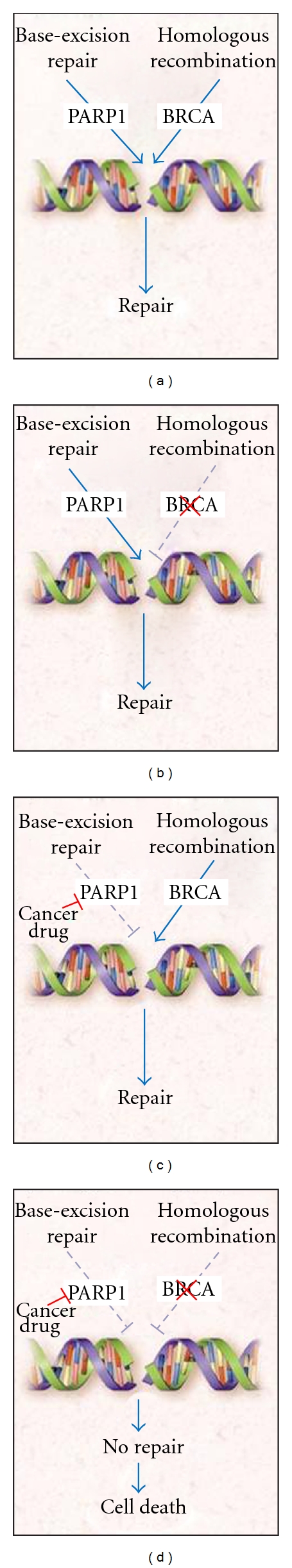
Depiction of BRCA mutations and PARP1 inhibitors blocking DNA repair and causing cell death [31]. Copyright ©  2009 Massachusetts Medical Society. All rights reserved.

Homologous recombination is mediated by several factors, including BRCA1, BRCA2, and RAD51 [26–28]. Cells deficient in functioning homologous recombination, such as ones with defective BRCA1 and/or BRCA2 genes, are forced into less precise repair pathways that make them more susceptible to cell death when overwhelmed with defects to repair [29]. These alternate pathways include nonhomologous end joining. The incorrect pairing of ends of DNA then possibly leads to genomic instability, ultimately ending in apoptosis (Figure 2). Interestingly, PARP is also involved in dsDNA repair in combination with nonhomologous end joining, so PARP inhibition also hinders the cell's other repair routes [24]. PARP1 inhibitors are being investigated as pharmacologic interventions for metastatic TNBC due to a theory of selectivity: if only BRCA-defective genes are terminated, then other cells that maintain a normal, functioning BRCA allele will not be killed by a PARP inhibitor. This synthetic lethality is being developed to create a new class of drugs that aim to efficiently kill cancer cells.

## 3. Current Therapeutic Strategy

Several PARP1 inhibitors are being studied at the clinical trial level, and this paper will focus specifically on iniparib, olaparib, and veliparib (Table 1, http://www.clinicaltrials.gov/). Results of an open-label phase II trial for iniparib (BSI-201, Sanofi-Aventis) combined with chemotherapy on metastatic TNBC patients were recently published [30]. This trial compared the use of gemcitabine and carboplatin alone versus those two agents and iniparib. The median progression-free survival increased when iniparib was added, from 3.6 to 5.9 months. The median overall survival was also significantly increased in the iniparib group, up to 12.3 months from 7.7 months. A complete or partial response was seen in 56% of patients receiving iniparib, while only 34% exhibited such a response in the gemcitabine/carboplatin arm. Common side effects seen amongst the 116 patients were nausea, fatigue, anemia, and neutropenia. It is notable that these side effects did not increase when iniparib was added to the regimen, suggesting that the side effects originate from gemcitabine and/or carboplatin.

A notable component of this study is that BRCA1/2 status was not assessed on the patients. Domagala et al. have claimed that 18% of BRCA1-associated cancers have low or no nuclear expression of PARP1 [32] and low PARP1 expression in 21% of triple-negative BRCA1-associated breast cancers [33]. When looking at cytoplasmic and nuclear PARP, another group has observed its presence in all intrinsic types of breast cancer, albeit with different frequencies [34]. There was a significant correlation between cytoplasmic and nuclear PARP in that study. Clearly, the expression pattern and full mechanism of PARP1 needs to be investigated to better understand if it will be an effective target for TNBC. 

At this year's meeting of the American Society of Clinical Oncology, O'Shaughnessy and colleagues presented their results of the phase III iniparib trial. This trial enrolled 519 women and again looked at gemcitabine and carboplatin versus the same regimen with added iniparib. The results did find an increase in progression-free survival amongst the iniparib/gemcitabine/carboplatin arm (5.1 versus 4.1, *P* = 0.027), but this did not achieve the prespecified criteria for significance (*P* = 0.01) [35]. A possible explanation behind the change in results from phase II to phase III is that the heterogenous nature of TNBC will continue to make finding a single agent problematic in treating all comers. By not stratifying the patients based on BRCA status or TNBC subtype, it leaves questions as to which patients will truly benefit from this drug and which have a genetic makeup that is not conducive to iniparib. Iniparib is continuing to be studied in other phase III clinical trials, including its effects on nonsmall cell lung cancer and ovarian cancer. Iniparib evidently is not being discontinued completely from breast cancer research; rather, the drug maker has continued with phase II trials analyzing different doses, schedules, and chemotherapy combinations.

Olaparib (AZD2281, AstraZeneca) is another PARP1 inhibitor that is being tested on various cancers, including breast. Preclinical models showed an increased selective potency for this compound [36]. The subsequent phase I trial revealed 400 mg twice daily to be the maximum dose. With a BRCA1- or BRCA2-defective cohort of 22 patients, antitumor efficacy was observed once the dosages reached 100 mg twice daily [37]. Results of a phase II trial detailed how olaparib is effective in breast cancer patients with a BRCA1 or BRCA2 mutation and advanced disease [38]. While admittedly not a flawless design, such as lacking randomization, the results showed promise. All patients in the study had locally advanced breast cancer (LABC) or metastatic breast cancer. For the TNBC and BRCA1/2 carrier patients in this cohort, twice daily 400 mg dosages of olaparib were more effective than twice daily 100 mg dosages when analyzing objective response (54% versus 25%) and progressive disease (15% versus 31%). These data were observed, but it must be noted that this trial was not designed or powered for this comparison. When looking at all of the women in the trial, 41% of the BRCA1- or BRCA2-mutated breast cancer patients had an objective response when assigned 400 mg twice-daily olaparib.

Despite these encouraging results, London-based drug maker AstraZeneca has decided to suspend olaparib prior to a phase III trial. AstraZeneca has shifted its olaparib focus to ovarian cancer and currently has a phase II trial to study its effects on that cancer type [39]. 

Veliparib (ABT-888, Abbot Laboratories) has been investigated as a single agent [40] and also has been shown to improve laboratory outcomes when paired with platinum agents and radiotherapy. Donawho et al. were able to show that 5 and 25 mg/kg/d of veliparib combined with cisplatin were significant in tumor regression of murine models compared to cisplatin alone [41]. 10 mg/kg/d of veliparib was also shown to be effective in combination with carboplatin when compared to carboplatin alone. In addition to improving the effectiveness of platinum agents on murine models of breast cancer, veliparib has shown to assist in radiation therapy. In mice, 3 Gy with added veliparib was significantly more effective in inducing early cellular senescence than just the radiation alone [42]. A phase II trial recently studied the effects of veliparib combined with temozolomide on metastatic breast cancer and included TNBC patients [43]. Of the 51 patients in the study, only 8 had a BRCA mutation. Progression-free survival was 5.5 months in the BRCA-mutated group versus 1.8 months for patients without a BRCA mutation. This suggests that veliparib might only be effective in patients carrying BRCA mutations.

## 4. Conclusion

TNBC is a clinical term used to describe women whose tumors lack expression of ER, PR, and HER-2. This subset of breast cancer partially fits into a molecular subtype known as basal-like breast cancer. Regardless of whether one looks at data through a TNBC or basal-like spectrum, the prognosis is worse compared to other subtypes. While there is no specific treatment regimen for TNBC patients, neoadjuvant therapy has been effective in achieving complete pathologic response (pCR) that subsequently correlates to improved outcome [7, 44]. TNBC patients who achieve pCR had similar overall survival rates to non-TNBC patients who achieved pCR. However, TNBC patients that did not reach pCR had a worse outcome compared to non-TNBC patients that did not reach pCR. 

Therapeutic options for TNBC have the potential to drastically increase in the near future. Combinations of platinum compounds for neoadjuvant therapy are being tested in various clinical trials. Epidermal growth factor receptors (EGFRs) are noted in 45–70% of TNBC [45, 46], resulting in EGFR antagonists such as cetuximab (Merck Serono) to be explored. Linderholm et al. noted VEGF to be increased in their TNBC patients compared to non-TNBC [47], and the antiangiogenic agent bevacizumab is being studied in combination with several chemotherapy agents in clinical trials. Still other emerging avenues for treatment include mammalian target of rapamycin (mTOR) inhibitors and SRC tyrosine kinase inhibitors.

Many potential therapeutic agents are in the pipeline in laboratories worldwide, but PARP inhibitors have the potential to alter the outcome of TNBC patients. In addition to iniparib, olaparib, and veliparib, there are more being constructed. These include CEP-9722 (Cephalon), INO-1001 (Genentech), PF-01367338 (Clovis/Pfizer), and MK-4827 (Merck). 

Several challenges must still be met to continue advancing PARP inhibitors. Most notably is the fact that recent trial data have landed huge blows to the momentum of PARP inhibitors for breast cancer. At the 2011 ASOC, it was announced that iniparib did not perform at its expected effectiveness in a phase III trial with metastatic TNBC patients. AstraZeneca has maintained an interest in PARP inhibitors, but is doing so through further trials in other organs, such as ovarian. Yet another complication that has emerged is resistance to PARP inhibitors that is being observed in the laboratory [48]. Norquist et al. recently reported to observe cell lines with BRCA1/2 restoration mutations exhibiting resistance to platinum therapy in patients with hereditary ovarian cancer. They also observed these restoration mutations to predict resistance to PARP inhibitors, but did not have a large sample size [49]. More research must be done on these compounds to prepare for these and other, unknown, complications.

It will be imperative to continue exploring the pathway connecting TNBC, basal-like breast cancer, and BRCA. There appears to be more questions to explore and compounds to test in the TNBC population with these therapeutics. Also, further testing is necessary to identify the optimal doses of not only the PARP inhibitor but also any combined chemotherapy. These key components of PARP inhibitor development will hopefully improve the quality of this class of cancer-fighting drugs and provide hope for patients currently facing such bleak diagnoses. 

## Figures and Tables

**Table 1:** Partial list of ongoing clinical trials for PARP inhibitors on TNBC.

Drug/company	Trial ID	Trial	Phase
Olaparib (AZD2281)/AstraZeneca	NCT01116648	Cediranib and olaparib	II
	NCT00647062	AZD2281 and carboplatin	I
	NCT00516724	In combination with carboplatin and/or paclitaxel	I
	NCT00707707	In combination with paclitaxel	I
	NCT00679783	In known BRCA/TNBC	II

Iniparib (BSI-201)/Sanofi-Aventis	NCT01173497	Iniparib + irinotecan	II
	NCT00813956	Neoadjuvant with gemcitabine and carboplatin	II
	NCT01045304	Metastatic with gemcitabine and carboplatin	II
	NCT01204125	Neoadjuvant with paclitaxel	II
	NCT01130259	In combination with gemcitabine and carboplatin	III

Veliparib (ABT-888)/Abbott	NCT01009788	With temozolomide	II
	NCT01104259	With cisplatin and vinorelbine ditartrate	I
	NCT01306032	With cyclophosphamide	II
	NCT01042379	I-SPY2 trial	II
	NCT01251874	With carboplatin	I

Data obtained from http://www.clinicaltrials.gov, June 15, 2011.

## References

[1] Perou CM, Sørile T, Eisen MB (2000). Molecular portraits of human breast tumours. *Nature*.

[2] Sørlie T, Perou CM, Tibshirani R (2001). Gene expression patterns of breast carcinomas distinguish tumor subclasses with clinical implications. *Proceedings of the National Academy of Sciences of the United States of America*.

[3] Sotiriou C, Neo SY, McShane LM (2003). Breast cancer classification and prognosis based on gene expression profiles from a population-based study. *Proceedings of the National Academy of Sciences of the United States of America*.

[4] Van’t Veer LJ, Dai H, Van de Vijver MJ (2002). Gene expression profiling predicts clinical outcome of breast cancer. *Nature*.

[5] Kwan ML, Kushi LH, Weltzien E (2009). Epidemiology of breast cancer subtypes in two prospective cohort studies of breast cancer survivors. *Breast Cancer Research*.

[6] Stark A, Kapke A, Schultz D, Brown R, Linden M, Raju U (2008). Advanced stages and poorly differentiated grade are associated with an increased risk of HER2/neu positive breast carcinoma only in White women: findings from a prospective cohort study of African-American and White-American women. *Breast Cancer Research and Treatment*.

[7] Rouzier R, Perou CM, Symmans WF (2005). Breast cancer molecular subtypes respond differently to preoperative chemotherapy. *Clinical Cancer Research*.

[8] Carey LA, Dees EC, Sawyer L (2007). The triple negative paradox: primary tumor chemosensitivity of breast cancer subtypes. *Clinical Cancer Research*.

[9] Bauer KR, Brown M, Cress RD, Parise CA, Caggiano V (2007). Descriptive analysis of estrogen receptor (ER)-negative, progesterone receptor (PR)-negative, and HER2-negative invasive breast cancer, the so-called triple-negative phenotype: a population-based study from the California Cancer Registry. *Cancer*.

[10] Millikan RC, Newman B, Tse CK (2008). Epidemiology of basal-like breast cancer. *Breast Cancer Research and Treatment*.

[11] Weigelt B, Baehner FL, Reis-Filho JS (2010). The contribution of gene expression profiling to breast cancer classification, prognostication and prediction: a retrospective of the last decade. *Journal of Pathology*.

[12] Bertucci F, Finetti P, Cervera N (2008). How basal are triple-negative breast cancers?. *International Journal of Cancer*.

[13] Wooster R, Weber BL (2003). Breast and ovarian cancer. *New England Journal of Medicine*.

[14] Turner NC, Reis-Filho JS, Russell AM (2007). BRCA1 dysfunction in sporadic basal-like breast cancer. *Oncogene*.

[15] Esteller M, Silva JM, Dominguez G (2000). Promoter hypermethylation and BRCA1 inactivation in sporadic breast and ovarian tumors. *Journal of the National Cancer Institute*.

[16] Rice JC, Ozcelik H, Maxeiner P, Andrulis I, Futscher BW (2000). Methylation of the BRCA1 promoter is associated with decreased BRCA1 mRNA levels in clinical breast cancer specimens. *Carcinogenesis*.

[17] Liu S, Ginestier C, Charafe-Jauffret E (2008). BRCA1 regulates human mammary stem/progenitor cell fate. *Proceedings of the National Academy of Sciences of the United States of America*.

[18] Foulkes WD, Stefansson IM, Chappuis PO (2003). Germline BRCA1 mutations and a basal epithelial phenotype in breast cancer. *Journal of the National Cancer Institute*.

[19] Lakhani SR, Reis-Filho JS, Fulford L (2005). Prediction of BRCA1 status in patients with breast cancer using estrogen receptor and basal phenotype. *Clinical Cancer Research*.

[20] Sørlie T, Tibshirani R, Parker J (2003). Repeated observation of breast tumor subtypes in independent gene expression data sets. *Proceedings of the National Academy of Sciences of the United States of America*.

[21] Rouleau M, Patel A, Hendzel MJ, Kaufmann SH, Poirier GG (2010). PARP inhibition: PARP1 and beyond. *Nature Reviews Cancer*.

[22] Kinsella TJ (2009). Understanding DNA damage response and DNA repair pathways: applications to more targeted cancer therapeutics. *Seminars in Oncology*.

[23] Löbrich M, Jeggo PA (2007). The impact of a negligent G2/M checkpoint on genomic instability and cancer induction. *Nature Reviews Cancer*.

[24] Saleh-Gohari N, Bryant HE, Schultz N, Parker KM, Cassel TN, Helleday T (2005). Spontaneous homologous recombination is induced by collapsed replication forks that are caused by endogenous DNA single-strand breaks. *Molecular and Cellular Biology*.

[25] Shrivastav M, De Haro LP, Nickoloff JA (2008). Regulation of DNA double-strand break repair pathway choice. *Cell Research*.

[26] Wong AKC, Pero R, Ormonde PA, Tavtigian SV, Bartel PL (1997). RAD51 interacts with the evolutionarily conserved BRC motifs in the human breast cancer susceptibility gene brca2. *Journal of Biological Chemistry*.

[27] Scully R, Chen J, Plug A (1997). Association of BRCA1 with Rad51 in mitotic and meiotic cells. *Cell*.

[28] Chen J, Silver DP, Walpita D (1998). Stable interaction between the products of the BRCA1 and BRCA2 tumor suppressor genes in mitotic and meiotic cells. *Molecular Cell*.

[29] Lord CJ, Ashworth A (2008). Targeted therapy for cancer using PARP inhibitors. *Current Opinion in Pharmacology*.

[30] O'Shaughnessy J, Osborne C, Pippen JE (2011). Iniparib plus chemotherapy in metastatic triple-negative breast cancer. *New England Journal of Medicine*.

[31] Iglehart JD (2009). Depiction of BRCA mutations and PARP1 inhibitors blocking DNA repair and causing cell death. *The New England Journal of Medicine*.

[32] Domagala P, Huzarski T, Lubinski J, Gugala K, Domagala W (2011). Immunophenotypic predictive profiling of BRCA1-associated breast cancer. *Virchows Archiv*.

[33] Domagala P, Huzarski T, Lubinski J, Gugala K, Domagala W (2011). PARP-1 expression in breast cancer including BRCA1-associated, triple negative and basal-like tumors: possible implications for PARP-1 inhibitor therapy. *Breast Cancer Research and Treatment*.

[34] Von Minckwitz G, Müller BM, Loibl S (2011). Cytoplasmic poly(adenosine diphosphate-ribose) polymerase expression is predictive and prognostic in patients with breast cancer treated with neoadjuvant chemotherapy. *Journal of Clinical Oncology*.

[35] O’Shaughnessy J, Schwartzberg LS, Danso MA, Rugo HS, Miller K (2011). A randomized phase III study of iniparib (BSI-201) in combination with gemcitabine/carboplatin (G/C) in metastatic triple negative breast cancer. *Journal of Clinical Oncology*.

[36] Farmer H, McCabe H, Lord CJ (2005). Targeting the DNA repair defect in BRCA mutant cells as a therapeutic strategy. *Nature*.

[37] Fong PC, Boss DS, Yap TA (2009). Inhibition of poly(ADP-ribose) polymerase in tumors from BRCA mutation carriers. *New England Journal of Medicine*.

[38] Tutt A, Robson M, Garber JE (2010). Oral poly(ADP-ribose) polymerase inhibitor olaparib in patients with BRCA1 or BRCA2 mutations and advanced breast cancer: a proof-of-concept trial. *The Lancet*.

[39] Guha M (2011). PARP inhibitors stumble in breast cancer. *Nature Biotechnology*.

[40] Yang SX, Kummar S, Steinberg SM (2009). Immunohistochemical detection of poly(ADP-ribose) polymerase inhibition by ABT-888 in patients with refractory solid tumors and lymphomas. *Cancer Biology and Therapy*.

[41] Donawho CK, Luo Y, Luo Y (2007). ABT-888, an orally active poly(ADP-ribose) polymerase inhibitor that potentiates DNA-damaging agents in preclinical tumor models. *Clinical Cancer Research*.

[42] Efimova EV, Mauceri HJ, Golden DW (2010). Poly(ADP-ribose) polymerase inhibitor induces accelerated senescence in irradiated breast cancer cells and tumors. *Cancer Research*.

[43] Isakoff SJ, Overmoyer B, Tung NM (2009). A phase II trial of the PARP inhibitor veliparib (ABT888) and temozolomide for metastatic breast cancer. *Journal of Clinical Oncology*.

[44] Liedtke C, Mazouni C, Hess KR (2008). Response to neoadjuvant therapy and long-term survival in patients with triple-negative breast cancer. *Journal of Clinical Oncology*.

[45] Nielsen TO, Hsu FD, Jensen K (2004). Immunohistochemical and clinical characterization of the basal-like subtype of invasive breast carcinoma. *Clinical Cancer Research*.

[46] Collins LC, Martyniak A, Kandel MJ (2009). Basal cytokeratin and epidermal growth factor receptor expression are not predictive of BRCA1 mutation status in women with triple-negative breast cancers. *American Journal of Surgical Pathology*.

[47] Linderholm BK, Hellborg H, Johansson U (2009). Significantly higher levels of vascular endothelial growth factor (VEGF) and shorter survival times for patients with primary operable triple-negative breast cancer. *Annals of Oncology*.

[48] Edwards SL, Brough R, Lord CJ (2008). Resistance to therapy caused by intragenic deletion in BRCA2. *Nature*.

[49] Norquist B, Wurz KA, Pennil CC, Garcia R, Gross J (2011). Secondary somatic mutations restoring BRCA1/2 predict clinical chemotherapy resistance in hereditary ovarian carcinomas. *Journal of Clinical Oncology*.

